# A Multi-Center, Prospective Observational Study to Investigate the Safety, Compliance, and Efficacy of Omethyl QTlet Soft Capsule

**DOI:** 10.3390/jcm11236949

**Published:** 2022-11-25

**Authors:** You-Jeong Ki, Sang-Jin Han, Tae-Joon Cha, Jae Hyuk Lee, Eui Kyo Seo, Jae Won Yang, Won Min Hwang, Dong Kyu Jin, Joo-Hyun Park, Han Young Ryu, Chang Gyu Park, Jun Hong Lee, Si Wan Choi, Eun Jeong Cho, Weon Kim

**Affiliations:** 1 Cardiovascular Division, Department of Internal Medicine, Kyung Hee University Hospital, Kyung Hee University, Seoul 02447, Republic of Korea; 2 Division of Cardiology, Department of Internal Medicine, Hallym University Sacred Heart Hospital, Anyang 14068, Republic of Korea; 3 Division of Cardiology, Kosin University Gospel Hospital, Busan 49267, Republic of Korea; 4 Division of Endocrinology, Department of Internal Medicine, Diabetes & Endocrinology Center, Myongji Hospital, Hanyang University Medical Center, Goyang 10475, Republic of Korea; 5 Department of Neurosurgery, Ewha Womans University School of Medicine, Seoul 07804, Republic of Korea; 6 Division of Nephrology, Department of Internal Medicine, Yonsei University Wonju College of Medicine, Wonju 26426, Republic of Korea; 7 Divsion of Nephrology, Department of Internal Medicine, College of Medicine, Konyang University, Daejeon 35365, Republic of Korea; 8 Division of Cardiology, Department of Internal Medicine, Soonchunhyang University Cheonan Hospital, Cheonan 31151, Republic of Korea; 9 Department of Family Medicine, Korea University Ansan Hospital, Korea University College of Medicine, Ansan 15355, Republic of Korea; 10 Department of Thoracic and Cardiovascular Surgery, Konyang University Hospital, Daejeon 35365, Republic of Korea; 11 Cardiovascular Center, Korea University Guro Hospital, Seoul 08308, Republic of Korea; 12 Department of Neurology, National Health Insurance Service Ilsan Hospital, Goyang 10444, Republic of Korea; 13 Division of Cardiology, Department of Internal Medicine, Chungnam National University Hospital, Chungnam National University School of Medicine, Daejeon 35015, Republic of Korea; 14 Division of Cardiology, Department of Internal Medicine, Heart Brain Hospital, Chung-Ang University Gwangmyeong Hospital, Chung-Ang University College of Medicine, Gwangmyeong 14353, Republic of Korea

**Keywords:** Omethyl QTlet soft capsule, hypertriglyceridemia, omega-3 fatty acids

## Abstract

Omega-3 fatty acids have been shown to be effective in lowering triglyceride (TG) levels; however, tolerability issues arise due to the large size of the pills. The purpose of this study was to examine the safety, compliance, and efficacy of Omethyl QTlet soft capsules (OQCs). This multi-center, prospective, observational study evaluated the safety, compliance, and efficacy of OQCs. Patients with hypertriglyceridemia with a history of omega-3 fatty acid intake were enrolled in this study and were prescribed OQCs (2 g–4 g/day) for eight weeks. All adverse events (AEs), adverse drug reactions (ADRs), and serious adverse events (SAEs) were recorded for safety evaluation. Adherence to treatment was assessed using questionnaires, and efficacy was assessed by changes in lipid and lipoprotein levels after eight weeks from baseline. The convenience of taking medication was analyzed for 580 patients, and the efficacy test was performed for 563 patients. The AE and ADR rates were 8.2% and 5.7%, respectively. There were only two SAEs. Of the patients, 55.8% responded that the OQC improved medication convenience, and mean changes in TG, total cholesterol, LDL-C, and non-HDL-C from baseline to eight weeks were −37.88 mg/dL, −11.56 mg/dL, −5.55 mg/dL, and −10.87 mg/dL, respectively (*p*-values < 0.001). In patients who had previously taken omega-3 fatty acids, OQCs showed safety and efficacy in lowering TG, and it was confirmed that compliance with medicine also improved compared to omega-3 fatty acids.

## 1. Introduction

Cardiovascular disease (CVD) is the leading cause of mortality worldwide. It is estimated that 17.9 million people died from CVDs in 2019, accounting for 32% of global deaths [1,2]. The prevalence of CVD, which is a major cause of rising medical costs, is continuously increasing. For this reason, appropriate management of chronic diseases, such as hypertension, diabetes mellitus, and dyslipidemia, is emphasized [1].

Notably, dyslipidemia is an important and modifiable risk factor for CVD [3] with a prevalence of 40% in adults over the age of 20 years in Korea (five out of ten men and three out of ten women) [4]. Hypertriglyceridemia is defined as a triglyceride (TG) level greater than 200 mg/dL and is more prevalent in men than women [4]. Although the fist-line goal in the treatment of dyslipidemia is to lower low-density lipoprotein cholesterol (LDL-C), hypertriglyceridemia is also a well-known independent risk factor associated with CVD [5,6,7].

**Figure 1:**
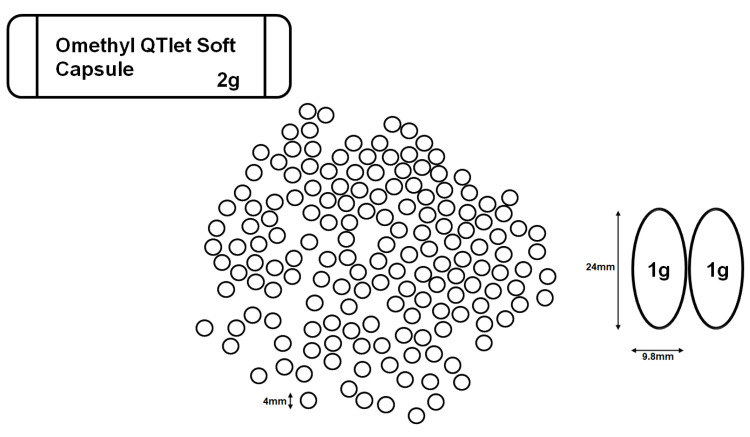
The Omethyl QTlet soft capsule is a generic drug that compensates for problems, such as dysphagia, by reducing the previous oval formulation with a long axis of 15 to 25 mm per 1 g to a formulation with a diameter of 4 mm at 2 g. Abbreviations: OQC, Omethyl QTlet soft capsule.

Omega-3 fatty acids have proven their TG-lowering effect with good safety, tolerability, and reduced drug interactions [8,9]. However, since the size of the pill is too large and it is often taken three to four times a day, the convenience of taking the medication is low. The Omethyl QTlet soft capsule (OQC) is a generic drug that compensates for problems, such as dysphagia, by reducing the previous oval formulation with a long axis of 15 to 25 mm per 1 g to a formulation with a diameter of 4 mm at 2 g (Figure 1). This study aimed to examine the safety, compliance, and efficacy of OQCs containing omega-3-acid ethyl ester 90 in Korean patients with hypertriglyceridemia.

## 2. Materials and Methods

### 2.1. Study Design

This eight-week prospective trial was conducted in 18 centers in South Korea. The study period was from 30 January 2020 through 11 June 2021. Patients with hypertriglyceridemia with a history of omega-3 fatty acid intake were enrolled in this study. Participants were required to meet the following criteria: (1) participants aged ≥19 years, (2) patients who had previously taken omega-3 fatty acids 90, (3) patients with hypertriglyceridemia who plan to take OQCs and have the following indications: (1) monotherapy for the treatment of hypertriglyceridemia; (2) combination therapy with statins for the treatment of combined hyperlipidemia (high cholesterol and high TG levels), (3) patients who plan to take the OQC for eight weeks, (4) participants that consented to written informed consent. The exclusion criteria were as follows: (1) hypersensitivity to ingredients in the OQC and (2) those who were judged unsuitable by researchers. Eligible patients were prescribed OQCs (2 g or 4 g per day) for eight weeks according to clinical judgement or clinical environment. The study protocol was approved by the institutional review board or ethics committee of each participating center, and all patients provided written informed consent.

### 2.2. Efficacy and Tolerability Assessments

The primary endpoint of this study was safety outcomes. Adverse events (AEs), adverse drug reactions (ADRs), and serious adverse events (SAEs) were collected regardless of whether they were predictable. The secondary endpoints were the convenience of drug administration and efficacy of changing the lipid and lipoprotein levels, including TGs, non-high-density lipoprotein cholesterol (non-HDL-C), total cholesterol, low-density lipoprotein cholesterol (LDL-C), and HDL-C, after eight weeks from baseline.

The convenience of administration was evaluated in terms of overall improvement, improvement in detailed items, and adherence to medication compared to previous omega-3 fatty acids. Compliance with treatment was assessed using patient-reported outcomes (PROs) based on questionnaires. A PRO is reported by the patients without the clinician interpreting the patient’s response related to health, quality of life, or functional status related to treatment [10]. To compare the degree of improvement in the convenience of taking medicine, patients who have taken omega-3 fatty acids in the past were enrolled, and improved convenience was assessed through a survey with seven degrees of response related to the overall improvement (very improved, improved, slightly improved, no change, slightly worse, worse, very bad). The answers of very improved, improved, and slightly improved were classified as “improved”, and those of slightly worse, worse, and very bad were classified as “worse”. To assess the improvement of details, symptoms related to taking medicine were assessed using the questions receiving the following responses: choke on, fishy smell, reflux, burp, nausea, and diarrhea. Six symptoms were evaluated on a five-point scale ranging from 0 to 4 points. The higher the score, the lower the convenience of taking the medicine.

Efficacy was assessed by changes in lipid and lipoprotein levels, including TGs, non-HDL-C, total cholesterol, LDL-C, and HDL-C, eight weeks after.

### 2.3. Statistical Analysis

The safety set was defined as patients enrolled in this study who took the drug at least once and were used in the analysis of safety endpoints. Other endpoints were first analyzed on an intention-to-treat basis (full analysis set [FAS]) followed by a per-protocol basis (per-protocol set [PPS]). The FAS included all enrolled patients taking the drug at least once. The FAS for the convenience of taking medicine or efficacy evaluation of medicine was analyzed for subjects who performed each convenience or efficacy evaluation questionnaire at least once. PPS was defined as patients completing the research without violation according to the research plan for subjects included in the FAS analysis. Patients who met the following criteria were excluded: (1) drop out, (2) violation of inclusion/exclusion criteria, (3) medication compliance of less than 80%, and (4) other serious violations. The main analysis was based on the FAS. Variables related to the safety tests were analyzed without replacing the missing values. In case of missing values in the evaluation variables for convenience and efficacy, the most recently obtained data during FAS analysis were analyzed using the last observation carried forward method as if it were obtained at the time point. However, if there was no test of convenience or efficacy after obtaining the baseline characteristics, the missing values were not replaced. In the per-protocol analysis, an alternative method for missing values was not considered. The safety analysis is based on the safety set. The number of cases and incidence rates for all AEs, ADRs, and SAEs are presented according to the system organ class (SOC) and preferred term (PT).

Regarding the degree of exposure, the mean ± standard deviation (SD) and median values are presented for the duration and dose of the drug under investigation. The total drug administration dose was obtained by multiplying the number of drug bags administered at 2 g per bag. Adherence to medication was obtained as a continuous variable and presented as the mean ± SD and median value. The efficacy endpoints were represented by changes in lipid and lipoprotein levels based on the FAS and PP. To analyze differences between pre- and post- values, paired t-test was performed for normally distributed data, whereas nonparametric Wilcoxon signed-rank tests were taken for non-normally distributed data after distribution normality was verified by the Kolmogorov–Smirnov test. Efficacy evaluation for subgroups was conducted as follows: (1) each dose of OQC (2 g/day or 4 g/day), (2) each dose of omega-3 fatty acids taken previously, and (3) previous use of omega-3 fatty acids at the time of screening (recently taken for eight weeks or more, recently taken for less than eight weeks, or no use in the last eight weeks).

## 3. Results

### 3.1. Demographic Characteristics

**Figure 2:**
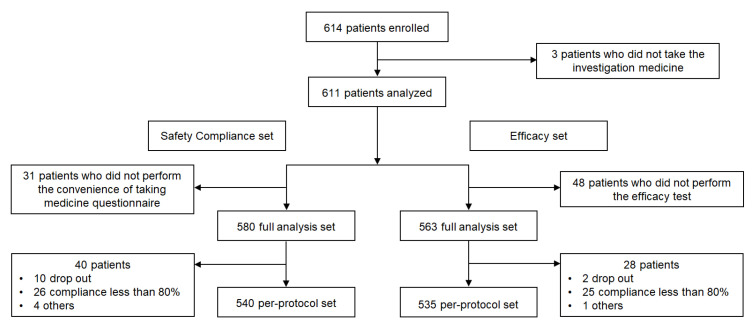
Flow diagram of participants. A total of 614 patients were enrolled, and the convenience of taking medication was analyzed for 580 patients among them, and an efficacy test was performed for 563 patients.

A total of 614 patients were enrolled of which three patients who did not take the investigational drug were excluded and 611 patients were analyzed. Regarding safety compliance, 580 patients were included in the full analysis, excluding 31 patients who did not complete the convenience questionnaire. A per-protocol analysis was performed on 540 subjects. In the efficacy evaluation, 48 patients did not undergo efficacy evaluation; therefore, 563 patients were included in the full analysis set, and 535 patients were included in the per-protocol set (Figure 2).

In the safety set, the mean administration duration was 58.5 days (SD: 8.8) and the mean total drug dose was 226.4 g (SD: 159.1). The baseline characteristics of the enrolled patients are shown in Table 1. The mean age was 60.6 years, 57.0% of the participants were males, and the mean body weight was 70.3 kg (SD: 13.5). More than half the patients (55.2%) had hypertension, and the prevalence of coronary artery disease was 29.6%.

### 3.2. Tolerability Evaluation

AEs and ADRs were reported in 8.2% (50/611 patients, 62 events) and 5.7% (35/611 patients, 44 events) of the patients, respectively. The rate of SAEs was 0.3% (2/611 patients, two events), and these events included melena and epididymitis requiring hospitalization or extending the admission duration. In addition, the rate of unexpected AEs was 3.1% (19/611 patients, 22 events). The incidence rate of unexpected ADRs was 2.0% (12/611 patients, 15 events), and there were no unexpected SAEs. The incidence rate of AEs leading to permanent discontinuation of medicine was 1.8% (11/611 patients, 12 events).

When classified into SOC and PT, gastrointestinal (GI) dysfunction (3.0%, 18/611 patients, 29 events) and dyspnea (1.3%, 8/611 patients, 8 events) were the most common AEs, respectively. Of the AEs, 88% were ‘mild’ (44/611 patients, 53 events), and there was one ‘severe’ AE, which was epididymitis.

When ADRs were classified as SOC, the most common organ dysfunction was GI dysfunction (2.5%, 15/611 patients, 17 events). According to the PT, ‘dyspnea’ was the most reported symptom (0.8%, 5/611 patients, five events). In SAE based on SOC, there was one GI disorder and one infection or infestation. One case of melena and one case of epididymitis were reported based on the PT.

The incidence of AEs was higher in the high dose group (4 g/day, 14.9%, 32/215 patients, 40 events) than in the low-dose group (2 g/day, 4.6%, 18/396 patients, 22 events). The most common AEs were dyspnea (2.8%, 6/215 patients, six events) in the high-dose group and nausea (0.8%, 3/396 patients, three events) in the low-dose group.

Thus, most AEs were classified as ‘mild’, and the most frequently identified events were dyspnea and dizziness. However, since the above symptoms are already approved AEs to medications containing omega-3 fatty acids, the OQC seems to be safe to use.

### 3.3. Improvement of Compliance in Taking Medicine

**Figure 3:**
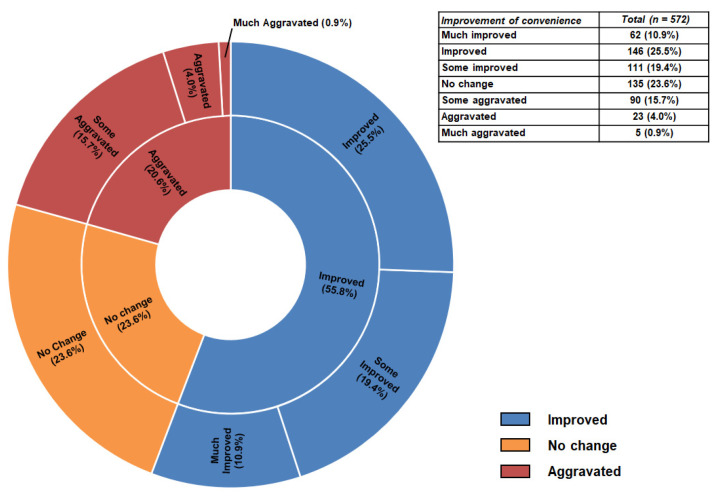
Overall improvement of convenience in full analysis set. In the questionnaire, 319 patients (55.8%) responded that the OQC improved medication convenience compared to the previous omega-3 fatty acids.

The overall improvement in the convenience of taking medication is shown in Figure 3. In the questionnaire, 319 patients (55.8%) responded that the OQC improved medication convenience compared to the previous omega-3 fatty acids. Table 2 shows the detailed improvements in the convenience of taking medicine. Various side effects related to omega-3 fatty acids were found to improve after taking OQCs except for a fishy smell. After eight weeks, the score significantly decreased, indicating that administration convenience improved except for the fish smell. At eight weeks, the compliance rate was 95.2% (SD: 12.5%).

In the subgroup analysis for each dose of OQC and each duration of omega-3 fatty acids previously taken, the overall and detailed improvement of convenience in taking medicine showed a similar tendency in the overall groups. In detail improvement, the symptoms of choke on, reflux, burp, and diarrhea showed improvement in all subgroups. Considering that the most common side effect of omega-3 fatty acids is GI symptoms, OQC is thought to reduce the digestive side effects of omega-3 fatty acids.

### 3.4. Efficacy Evaluation

**Figure 4:**
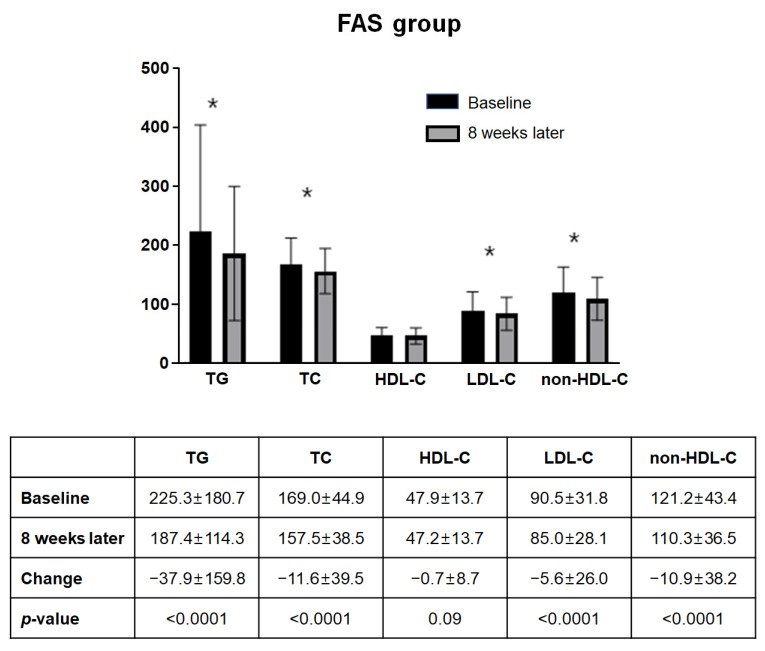
Change of lipid profile in FAS (*n* = 563). Among the 563 subjects included in the FAS, the lipid panel at eight weeks of administration showed a statistically significant decrease except for HDL-C compared to baseline. Abbreviations: FAS, full-analysis set; HDL-C, high density lipoprotein cholesterol; LDL-C, low density lipoprotein cholesterol; non-HDL-C, non-high density lipoprotein cholesterol; TC, total cholesterol; TG, triglyceride. * *p*-Value < 0.001.

**Figure 5:**
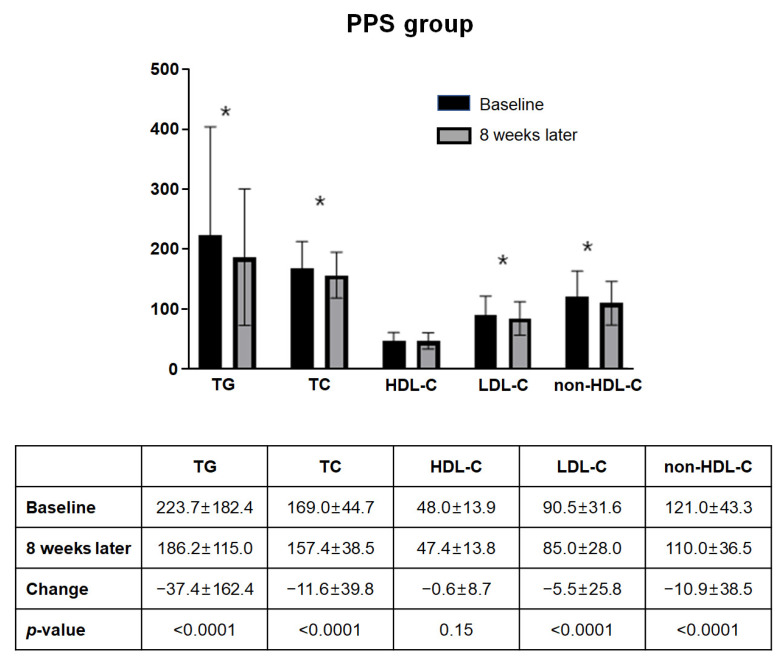
Change of lipid profile in PPS (*n* = 535). Among the 535 subjects included in the PPS, the lipid panel at eight weeks of administration showed a statistically significant decrease except for HDL-C compared to baseline. Abbreviations: HDL-C, high density lipoprotein cholesterol; LDL-C, low density lipoprotein cholesterol; non-HDL-C, non-high density lipoprotein cholesterol; PPS, per-protocol set; TC, total cholesterol; TG, triglyceride. * *p*-Value < 0.001.

Figure 4 summarizes the changes in lipid and lipoprotein levels in FAS. Among the 563 subjects included in the FAS, the lipid panel at eight weeks of administration showed a statistically significant decrease except for HDL-C compared to baseline. The mean changes in TG, total cholesterol, LDL-C, and non-HDL-C from baseline to eight weeks were −37.9 mg/dL (±159.8 mg/dL), −11.6 mg/dL (±39.5 mg/dL), −5.6 mg/dL (±26.0 mg/dL), and −10.9 mg/dL (±38.2 mg/dL), respectively (all *p*-Values <0.001). A similar tendency was observed in the PPS analysis (Figure 5). Both FAS and PPS analyses showed significant reductions in TG, total cholesterol, and non-HDL-C levels in all subgroups.

## 4. Discussion

The important points of this study were as follows. (1) Reported adverse reactions during use of OQCs were mostly ‘mild’. The most frequently reported side effects were dyspnea and dizziness. Since these symptoms are adverse symptoms reflected in the prior approval of medication containing omega-3-acid ethyl ester 90 as the main ingredient, the OQC can be used relatively safely. (2) OQC improved the convenience of taking in 55% of the patients compared to omega-3-acid ethyl ester 90 and improved convenience in detail. (3) In terms of efficacy, all lipid and lipoprotein levels except HDL-C decreased after eight weeks of administration of the OQC. This study confirmed the safety, compliance, and efficacy of OQCs in patients with hypertriglyceridemia who were administered omega-3-acid ethyl ester 90.

Although the first goal of dyslipidemia treatment is to reduce LDL-C, hypertriglyceridemia is also known as an independent risk factor of CVD because of the role of TG-rich lipoproteins in atherothrombosis [5,6,7]. In the postprandial status, dietary lipids are transported from the intestine to peripheral tissues via plasma lipoproteins called chylomicrons. In the capillary beds of peripheral tissues, chylomicron triglycerides are lipolyzed by the enzyme, lipoprotein lipase (LPL), allowing the delivery of free fatty acids to the cells. During this process, TG-rich lipoproteins lose the TG and C apolipoproteins and gain cholesteryl ester and apolipoprotein E (apo E) through the action of cholesteryl ester transfer protein. These particles become chylomicron remnants [11,12]. Accumulation of the remnants creates a proinflammatory and oxidizing environment that can enhance adhesion molecule expression, foam cell formation, and smooth muscle cytotoxicity [11]. The treatment of choice for dyslipidemia is statins, which effectively lower LDL-C but not TG. Hypertriglyceridemia is defined as TG levels ≥200 mg/dL, and the prevalence of hypertriglyceridemia was 22.4% in men and 9.7% in women in Korea [4]. Lifestyle modification and medication therapy were the main treatments for hypertriglyceridemia. Alcohol cessation, reduction of body weight, and exercise are recommended for the treatment of hypertriglyceridemia, and available treatment options for reducing TG levels include omega-3 fatty acids and fibrates [9,13]. The TG-lowering mechanisms of omega-3 is related to the effect of reducing hepatic production and secretion of VLDL and VLDL apo B particles, effects on plasma lipolytic activity through LPL-mediated clearance, and stimulation of beta-oxidation of other fatty acids in the liver [14]. In our study, the mean change in TG from baseline to eight weeks was −37.88 mg/dL. According to the current guideline, the primary treatment goal is to lower the LDL-C level below the target level according to the CV risk, and the secondary goal is to lower the non-HDL-C and apolipoprotein B levels [3].

Existing studies have observed that high LDL-C is associated with CVD; therefore, therapeutic strategies have primarily focused on LDL-C [15,16,17]. However, it is known that a residual CVD risk of 60–70% remains despite lowering LDL-C levels below 70 mg/dL, which makes us think about the role of TG on CVD risk [18,19]. In 29 published prospective studies, reports showed that the combined odds ratio for coronary heart disease (CHD) was 1.7 (95% CI, 1.6 to 1.9) in patients in the top third TG level compared with those in the bottom third TG level after adjusting several established risk factors [5]. In a sub-study of the PROVE IT-TIMI 22 trial, on-treatment TG < 150 mg/dL was associated with a lower risk of recurrent CVD events, which was independent of the level of LDL-C [19]. Even after adjusting LDL-C levels, for every 10 mg/dL decline in TG, there was a 1.6% decrease in composite cardiovascular (CV) outcomes. The FIELD and ACCORD lipid studies investigated fibrate-added therapy compared to statin monotherapy or placebo in patients with diabetes mellitus (DM), and fenofibrate did not significantly reduce composite CV events [13,20]. However, there was an insignificant heterogeneity treatment effect in the patients with TG levels ≥204 mg/dL and HDL-C levels ≤34 mg/dL compared to all other patients in the ACCORD lipid study [13]. Although not statistically significant, the incidence of primary outcomes was lower in the group with both a high baseline TG level and a low baseline HDL-C level (*p* for interaction = 0.06), suggesting a possible beneficial effect in this group. Fibrates also reduced total CV events, mainly through the prevention of non-fatal myocardial infarction (MI) and coronary revascularization [20]. Therefore, high-risk patients with persistent elevated TG levels despite prescribing statins should be treated with other TG-lowering agents. Available pharmacologic methods include statins, fibrates, PCSK9 inhibitors, and omega-3-fatty acids.

The omega-3 fatty acids eicosatetraenoic acid (EPA) and docosahexaenoic acid (DHA) lower TG levels by reducing the number of TG transportation lipoproteins, secondary to reducing hepatic very-low-density lipoprotein cholesterol production [21]. The TG lowering effect of omega-3 fatty acids has been demonstrated in the EVOLVE I and II trials [22,23]. There have been several studies on the CV benefits as well as the TG lowering effects of omega-3 fatty acids. In patients surviving recent MIs, the group receiving n-3 polyunsaturated fatty acids (PUFAs) (1 g daily) showed a significantly lower risk of the primary endpoint (death, MI, or stroke) than the control group [24]. The JELIS study showed that 1800 mg of EPA treatment reduced the frequency of major coronary events [25]. The beneficial effects of EPA were similar in both primary prevention and secondary prevention of CV risk. A recent REDUCE-IT trial demonstrated that 4 g of EPA significantly reduced the risk of ischemia events by approximately 25% compared to placebo in patients with established CV disease or diabetes who maintained TG levels between 135 and 499 mg/dL [26]. Based on these studies, the guideline suggested that n-3 PUFAs should be considered as a combination therapy in addition to statins in high-risk patients with high TG levels [3,27,28]. However, a Cochrane meta-analysis, including 112,059 people from 79 trials, showed no effect of n-3 fatty acids on mortality and CV events and showed little difference in CHD events (relative ratio: 0.93, 95% CI: 0.88–0.97). Furthermore, a recent ASCEND trial demonstrated that DM patients without atherosclerotic CV disease who received 1 g of n-3 fatty acids showed no significant difference in serious vascular events compared to the placebo group [29]. Therefore, high doses of n-3 fatty acids are required for their beneficial effects on CV disease.

Non-adherence to CV mediation is associated with an increase in CV events, and a meta-analysis has shown that a significant proportion of all CV events (~9% in Europe) are attributable to poor adherence to CV medications. The relative risks (95% CI) of development of CV disease in those with good vs. poor (<80%) adherence were 0.85 (0.81–0.89) for statins [30]. To maximize the effect on CV disease, high doses of n-3 fatty acid are required, but the existing omega-3 pills are long oval and bulky, making them inconvenient for patients to take. Naturally, adherence to medication is affected by the size of the pill and number of medications taken. The OQC is composed of small circles with a diameter of 4 mm so that 2 g can be taken per one sachet (Figure 1). Based on the results of a previous study, the OQC is safe, easy to take, and effective in lowering TG levels; therefore, it is expected to improve medication compliance in patients with hypertriglyceridemia.

Our study has potential limitations. First, the duration of treatment was short (eight weeks). Although further studies related to long-term usage are needed, this study found that OQCs effectively reduced TG and non-HDL-C levels even after eight weeks of treatment. Second, this study was conducted only on a Korean population. Therefore, we should be cautious in extrapolating these results to other ethnicities. Third, no special run-in period was set for the enrolled patients, and all patients who had ever taken omega-3 fatty acids were enrolled regardless of the duration, amount, or status of taking medicine. This may be a limitation of the study; however, it may be more practical because it reflects the real-world practice of changing medicine prescriptions. Fourth, our study did not directly compare omega-3 fatty acids and OQCs so we could not confirm the efficacy compared to omega-3 fatty acids. However, our study is meaningful in showing the medication compliance, safety, and efficacy of OQCs. Lastly, omega-3 PUFAs are known to be effective in reducing platelet aggregation, coagulation, and thrombosis [31,32], but our database did not allow us to analyze the effect of OQCs on platelets or thrombosis. Long term study on the anticoagulant activity of omega-3 fatty acids will be needed.

## 5. Conclusions

In patients who had previously taken omega-3 fatty acids, OQCs showed safety and efficacy in lowering TG, and it was confirmed that compliance with medication also improved compared to omega-3 fatty acids. Patients have the advantage of taking 2 g of the OQC per sachet compared to omega-3 fatty acids, which are difficult to ingest in the form of a large pill. Thus, OQCs are effective and well-tolerated in patients with hypertriglyceridemia.

## Figures and Tables

**Table 1:** Demography of study populations.

Characteristics	Total (*n* = 614)
Age	60.6 ± 11.9
Age < 60	280 (45.6%)
Age 60–80	304 (49.5%)
Age ≥ 80	30 (4.9%)
Male	350 (57.0%)
Height, cm	163.7 ± 8.8
Body weight, kg	70.3 ± 13.5
Hypertension	339 (55.2%)
Diabetes	67 (10.9%)
Coronary artery disease	182 (29.6%)
Stroke	470 (7.7%)
Indication of omega-3 fatty acids	
Monotherapy for treatment hypertriglyceridemia	154 (25.1%)
Combination therapy with statins for treatment of combined hyperlipidemia	364 (59.3%)
Combination therapy with statins in combined hyperlipidemia with uncontrolled TG	95 (15.5%)
Previous use of statin or ezetimibe	436 (71.0%)

Abbreviations: TG, triglyceride.

**Table 2:** Detail improvement of convenience in full analysis set.

	Total (*n* = 572)	Score Change after Treatment
**Total Score**		
Baseline		
Number of subjects	580	
Mean ± SD	3.55 ± 4.10	
Median (Min, Max)	2 (0, 20)	
After treatment		
Number of subjects	572	
Mean ± SD	2.27 ± 3.06	−1.28 ± 4.14 *
Median (Min, Max)	1 (0, 24)	
**Choke on**		
Baseline		
Number of subjects	580	
Mean ± SD	1.17 ± 1.22	
Median (Min, Max)	1 (0, 4)	
After treatment		
Number of subjects	572	
Mean ± SD	0.47 ± 0.91	−0.69 ± 1.40 *
Median (Min, Max)	0 (0, 4)	
**Fishy smell**		
Baseline		
Number of subjects	580	
Mean ± SD	0.75 ± 1.13	
Median (Min, Max)	0 (0, 4)	
After treatment		
Number of subjects	572	
Mean ± SD	0.96 ± 1.18	0.20 ± 1.40 *
Median (Min, Max)	1 (0, 4)	
**Reflux**		
Baseline		
Number of subjects	580	
Mean ± SD	0.55 ± 0.95	
Median (Min, Max)	0 (0, 4)	
After treatment		
Number of subjects	572	
Mean ± SD	0.25 ± 0.67	−0.30 ± 0.95 *
Median (Min, Max)	0 (0, 4)	
**Burp**		
Baseline		
Number of subjects	580	
Mean ± SD	0.49 ± 0.89	
Median (Min, Max)	0 (0, 4)	
After treatment		
Number of subjects	572	
Mean ± SD	0.25 ± 0.65	−0.23 ± 1.01 *
Median (Min, Max)	0 (0, 4)	
**Nausea**		
Baseline		
Number of subjects	580	
Mean ± SD	0.34 ± 0.76	
Median (Min, Max)	0 (0, 4)	
After treatment		
Number of subjects	572	
Mean ± SD	0.22 ± 0.57	−0.14 ± 0.80 *
Median (Min, Max)	0 (0, 4)	
**Diarrhea**		
Baseline		
Number of subjects	580	
Mean ± SD	0.26 ± 0.64	
Median (Min, Max)	0 (0, 4)	
After treatment		
Number of subjects	572	
Mean ± SD	0.14 ± 0.49	−0.12 ± 0.68 *
Median (Min, Max)	0 (0, 4)	

Abbreviations: SD, standard deviation. * *p*-value < 0.001.

## Data Availability

Data are available upon written request.
